# Investigation of Acid Tolerance Mechanism of *Acetobacter pasteurianus* under Different Concentrations of Substrate Acetic Acid Based on 4D Label-Free Proteomic Analysis

**DOI:** 10.3390/foods12244471

**Published:** 2023-12-13

**Authors:** Tian Li, Xinwei Wang, Chunyan Li, Qingquan Fu, Xuewei Shi, Bin Wang

**Affiliations:** Food College, Shihezi University, Shihezi 832000, China

**Keywords:** *Acetobacter pasteurianus*, 4D label-free proteomic, substrate acetic acid, acid resistant

## Abstract

*Acetobacter pasteurianus* is always used to brew vinegar because of its ability of producing and tolerating a high concentration of acetic acid. During vinegar fermentation, initial acetic acid contributes to acetic acid accumulation, which varies with initial concentrations. In this study, to investigate the mechanisms of tolerating and producing acetic acid of *Acetobacter pasteurianus* under different concentrations of substrate acetic acid, four-dimensional label-free proteomic technology has been used to analyze the protein profiles of *Acetobacter pasteurianus* at different growth stages (the lag and exponential phases) and different substrate acetic acid concentrations (0%, 3%, and 6%). A total of 2093 proteins were quantified in this study. The differentially expressed proteins were majorly involved in gene ontology terms of metabolic processes, cellular metabolic processes, and substance binding. Under acetic acid stress, strains might attenuate the toxicity of acetic acid by intensifying fatty acid metabolism, weakening the tricarboxylic acid cycle, glycerophospholipid and energy metabolism during the lag phase, while strains might promote the assimilation of acetic acid and inter-conversion of substances during the exponential phase by enhancing the tricarboxylic acid cycle, glycolysis, pyruvate, and energy metabolism to produce and tolerate acid. Besides, cell cycle regulation and protein translation might be potential acid tolerance pathways under high acid stress. The result contributes to the exploration of new potential acid tolerance mechanisms in *Acetobacter pasteurianus* from four-dimensional label-free relative quantitative proteomics analysis.

## 1. Introduction

Acetic acid bacteria (AAB) are a group of specialized aerobic Gram-negative bacteria capable of oxidizing sugars and alcohols to saccharic and carboxylic acids [1]. Currently, 19 genera and 92 species of AAB have been reported [2]. The AAB are named for their ability to oxidize ethanol to produce acetic acid, but the reported AAB that can be used to make vinegar is mainly from the genera *Komagataeibacter* and *Acetobacter*, such as *Komagataeibacter europaeus* and *Acetobacter pasteurianus* [2,3]. In China, *Acetobacter pasteurianus* (mainly *Acetobacter pasteurianus* CICC 20001 and CGMCC 1.41) is widely used in the brewing of fruit and grain vinegar and can produce and tolerate higher concentrations of acetic acid [2].

During acetic acid fermentation, AAB oxidize ethanol to acetic acid under aerobic conditions, and AAB are exposed to higher concentrations of ethanol and acetic acid [4,5]. This environment is lethal to most microorganisms, and AAB also produces large amounts of acetic acid in this environment [6]. In the face of an acidic environment with increasing concentrations of acetic acid, AAB can prevent the entry of acetic acid into the cell or reduce the intracellular concentration of acetic acid by means of cell membrane alteration [7], protection and repair of macromolecules [8] and two-component systems [9,10], acetic acid assimilation [11], proton motive force-dependent efflux system [12], ATP-binding cassette (ABC) transporter AatA [13], 2-methyl citrate cycle [11], toxin-antitoxin HicAB [14], and so on, thereby attenuating the toxic effects of acetic acid on AAB.

In the process of acetic acid fermentation, starting acetic acid fermentation with a certain concentration of acetic acid can accelerate the rate of acid production as well as the final accumulation of acetic acid [4]. It had been demonstrated that the addition of 0.5% or 1% of substrate acetic acid could significantly increase the rate of acetic acid production by acetic acid bacteria, and the acetic acid production capacity of acetic acid bacteria showed a large difference under different concentrations of substrate acetic acid [4]. The molecular mechanism of this difference needs to be further studied. The response of prokaryotes to acid changes is manifested at multiple levels, from the genetic level to the phenotypic level, of which the protein level is a key link. Proteomics is essentially a large-scale study of the characteristics of proteins under different conditions, including protein expression levels, post-translational modifications, protein–protein interactions, and so on, so as to obtain a holistic and comprehensive understanding at the protein level of disease mechanisms, cellular metabolism, and other processes [15]. Earlier, the expression of proteins under different concentrations of acetic acid and ethanol treatments was investigated using the two-dimension difference gel electrophoresis (2D-DIGE) technique, from which 2000 protein spots were detected [16]. In addition, 1224 proteins were quantified during the production of high acetic acid by *Acetobacter pasteurianus* using iTRAQ-dependent quantitative proteomic analysis [17]. However, these methods have certain limitations of their own. In recent years, with the continuous development of proteomics technology, label-free quantification techniques that rely on isotope labeling have emerged and are frequently used [18,19]. In four-dimensional (4D) label-free quantitative proteomics, mass spectrometry identifies and quantifies peptide ions based on the properties of four dimensions: retention time, mass charge ratio, ion intensity, and ion mobility [20]. It is widely used in the proteomics analysis of various biological samples, such as *Azotobacter chroococcum* [21], *Escherichia coli* [19], and Grape [22], due to its high sensitivity and wide detection range.

The addition of the substrate acetic acid affects the rate of production and accumulation of acetic acid by AAB, which varies with the initial concentration of acetic acid. Although some scholars have found that the addition of certain substrate acids promotes acetic acid fermentation, the molecular mechanism of acid production and acid tolerance metabolism of AAB at different concentrations of substrate acids initiating acetic acid fermentation is still unclear [4]. In order to investigate the molecular mechanism of acetic acid production and acid tolerance affected by different concentrations of substrate acid, in this study, we used a 4D label-free quantitative proteomics approach to analyze the differences in protein profiles of *Acetobacter pasteurianus* CICC 20001 under different growth stages (the lag and exponential phases) when acetic acid fermentation was initiated by different concentrations of substrate acetic acid (0%, 3% and 6%). The results will help us dig deeper into the potential molecular mechanisms of acid production and acid resistance of *Acetobacter pasteurianus* under different concentrations of acetic acid and also provide some theoretical references for the production of high-acidity vinegar.

## 2. Materials and Methods

### 2.1. Strains Cultivation, Collection and Sampling

The type strain *Acetobacter pasteurianus* CICC 20001 was purchased from the China Center of Industrial Culture Collection (CICC) and stored lyophilized in the laboratory. Involved medium refers to GY (10 g/L glucose and 1 g/L yeast extract) medium and GYP (1 g/L glucose, 5 g/L yeast extract and 2 g/L peptone) medium (glucose was purchased from Sheng’ao Chemical Reagents Co., Ltd., Tianjin, China; yeast extract and peptone were purchased from Aobox, Beijing, China). GYP1, GYP2, and GYP3 refer to GYP medium with 0%, 3% and 6% acetic acid (*v*/*v*), respectively, while 3% ethanol (*v*/*v*) was added to each medium (acetic acid and ethanol were purchased from Sinopharm Chemical Reagent Co., Ltd., Shanghai, China). The lyophilized culture was activated in 100 mL GY medium with 3% ethanol (*v*/*v*) contained in a 500 mL Erlenmeyer flask (170 rpm and 30 °C for 24 h in a shaker; Shaker, Zhicheng, Shanghai, China). The 10 mL activated strain was inoculated in 100 mL GYP medium with 3% ethanol (*v*/*v*) contained in a 500 mL Erlenmeyer flask under the condition of 170 rpm at 30 °C in a shaker. When the exponential phase (OD_600_ nm > 0.5; Spectrophotometer, Yoke instrument, Shanghai, China) was reached, 10 mL of the culture was then inoculated in 100 mL of GYP1, GYP2, and GYP3 media contained in the 500 mL Erlenmeyer flask (170 rpm and 30 °C in a shaker). The acidity in the cultures was determined by titration using 0.1 M NaOH (Macklin, Shanghai, China) with phenolphthalein as an indicator. When the sampling points were reached, 100 mL of cultures were immediately centrifuged at 8000 rpm below 4 °C for 10 min. The concentrated culture was immediately frozen in liquid nitrogen and then stored at −80 °C for analysis. Cell samples harvested at the lag (labeled ‘L’) and exponential periods (labeled ‘E’) were used for proteome analysis. The samples were designated as P0-L, P3-L, P6-L, P0-E, P3-E, and P6-E, respectively. The P0-L and P0-E samples were used as the controls, and others were regarded as the experimental groups in the proteomic analysis. Each treatment was repeated three times.

### 2.2. Proteomic Analysis

#### 2.2.1. Protein Extraction and Trypsin Digestion

The appropriate amounts of protein samples were placed in a liquid nitrogen pre-cooled mortar and ground well with liquid nitrogen until powder. Four times the volume of lysis buffer (8 M urea, 1% protease inhibitor) was added to the sample powder and the samples were sonicated three times on ice using a high intensity ultrasonic processor (Scientz, Ningbo, China). The supernatant was collected by centrifugation at 12,000× *g* for 10 min at 4 °C and protein concentration was determined using a bicinchoninic acid (BCA) assay kit (Beyotime, Shanghai, China) according to the manufacturer’s instructions. An equal amount of each sample protein was taken for enzymatic digestion and the volume was adjusted to consistency with the lysate. The protein samples were added to a one-fold volume of pre-cooled acetone, vortexed and mixed, then a four-fold volume of pre-cooled acetone was added and precipitated at −20 °C for 2 h. The precipitate was collected at 4500 g for 5 min and then washed twice with pre-cooled acetone. The air-dried precipitates were added to a final concentration of 200 mM triethylammonium bicarbonate, and the precipitates were sonicated to break them up, after which a 1:50 ratio (protease: protein, m/m) of trypsin was added and digested overnight. Dithiothreitol was added to a final concentration of 5 mM and the reaction was carried out at 56 °C for 30 min, following which iodoacetamide was added to a final concentration of 11 mM and alkylated for 15 min at room temperature in the dark [23].

#### 2.2.2. Liquid Chromatography–Tandem Mass Spectrometry (LC-MS/MS) Analysis

The tryptic peptides were solubilized with liquid chromatography mobile phase A and separated using a NanoElute ultra-high performance liquid chromatography (UHPLC) system (Bruker Daltonics, Germany). Mobile phase A was an aqueous solution containing 0.1% formic acid and 2% acetonitrile, mobile phase B was a solution containing 0.1% formic acid and 100% acetonitrile. The liquid phase gradient was set as follows: 0–70 min, 6–24% B; 70–84 min, 24–35% B; 84–87 min, 35–80% B; 87–90 min, 80% B. The flow rate was maintained at 450 nL/min. The peptides were injected into a capillary ion source for ionization and then analyzed by timsTOF Pro mass spectrometry (Bruker Daltonics, Germany), and the ion source voltage was set to 1.75 kV. Both the peptide parent ion and its secondary fragments were detected and analyzed using high-resolution TOF. The secondary mass spectrometry scanning range was set to 100–1700 *m*/*z*, and the data acquisition used was parallel accumulation serial fragmentation (PASEF) mode [22]. A primary mass spectrum was acquired, followed by 10 PASEF mode acquisitions of secondary spectra with parent ion charges in the range of 0–5, and the dynamic exclusion time of the tandem mass spectral scans was set to 30 s to avoid repeated scans of the parent ions [22].

#### 2.2.3. Protein Identification and Bioinformatics Analysis

The raw data were analyzed using the Maxquant software (v1.6.15.0) to identify and quantify the proteins. Trypsin/P was designated as a cleavage enzyme permitting up to two missing cleavages [23]. The primary parent ion mass error tolerance was set to 20 ppm for both the first search and the main search. The mass error tolerance for secondary fragment ions was also set to 20 ppm. The false discovery rate (FDR) for protein identification and peptide-spectrum match (PSM) identification were set to 1% [24]. The identified proteins need to contain at least one unique peptide segment. Differentially expressed proteins (DEPs) in samples were screened based on fold change (FC) and significance levels (*p* value). In our study, the screening criteria for DEPs were FC > 1.5 (up-regulated) or <0.67 (down-regulated), *p* value < 0.05. The DEPs were analyzed for protein structural domain annotation and subcellular structure prediction based on the Pfam database and the corresponding PfamScan tool and PSORTb software (v3.0), respectively. All DEPs in the samples were analyzed for Gene Ontology (GO), Cluster of Homologous Groups (COG) and Kyoto Encyclopedia of Genes and Genomes (KEGG) annotation. The protein–protein interactions (PPI) were constructed by Cytoscape 3.7.1 software based on predictions in the STRING server (http://string.embl.de/ (accessed on 15 October 2023). Protein structural domain enrichment, GO enrichment, and KEGG pathway enrichment significance analysis of DEPs using Fisher’s exact test.

### 2.3. Statistical Analysis

The result was analyzed by the analysis of variance (ANOVA) using the statistical program SPSS 25.0 (SPSS Inc., Chicago, IL, USA). The statistical significance was applied at the level of *p* < 0.05. The bar charts were plotted by origin 2023. Principal component analysis (PCA) was used to evaluate the differences in protein expression under different concentrations of substrate acetic acid. The illustration of relevant metabolic pathways and regulation were drawn using EdrawMax 9.1 soft.

## 3. Results

### 3.1. Overview of Acetobacter pasteurianus under Different Concentrations of Substrate Acetic Acid

**Figure 1:**
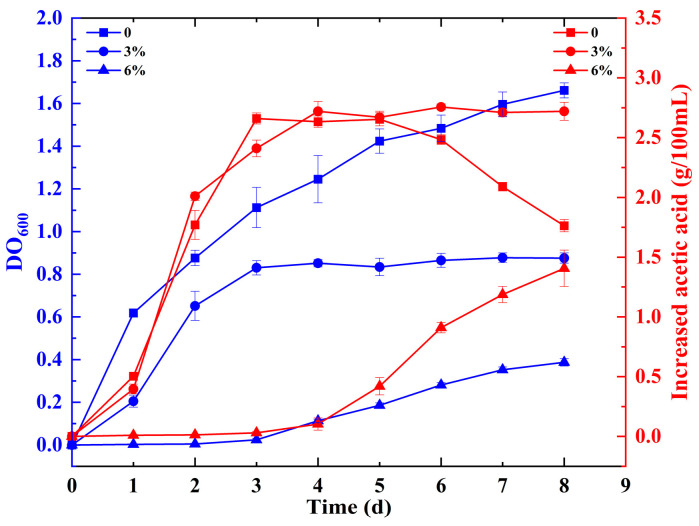
Growth and acidity of *Acetobacter pasteurianus* at different concentrations of substrate acetic acid.

For the purpose of this study, *Acetobacter pasteurianus* CICC 20001 was cultured in GYP1, GYP2, and GYP3 media, respectively. The growth (measured in OD_600_ nm) and acidity distribution in each culture are depicted in Figure 1. In addition, the growth of *Acetobacter pasteurianus* in GYP medium over a period of 24 h was shown in Table S1.

The cultures in GYP1 and GYP2 had a shorter lag phase. The GYP1 cultures grew continuously throughout the cultivation and reached an exponential phase on the second day of fermentation, reaching an OD_600_ value of about 0.8, and their increased acidity was 1.77 g/100 mL. However, the increased acidity started to show a decreasing trend at the fifth day of fermentation, which could be caused by the peroxidation of acetic acid [11]. The GYP2 cultures reached the exponential phase on the second day, reaching an OD_600_ value of about 0.6, and their increased acidity was 2.01 g/100 mL. Unlike the cultures in GYP3, which had a lag phase of three days and reached the exponential phase of six days, their increased acidity was 0.91 g/100 mL at the exponential phase. For proteomics sampling, the experimental samples were collected when fermentation in GYP medium entered the lag phase (P0-L, cultivated for 6 h in GYP1, increased acidity of 0.02 g/100 mL; P3-L, cultivated for 6 h in GYP2, increased acidity of 0.01 g/100 mL; P6, cultivated for 6 h in GYP3, increased acidity of 0.02 g/100 mL) and exponential phase (P0-E, cultivated for two days in GYP1, increased acidity of 1.77 g/100 mL; P3-E, cultivated for two days in GYP2, increased acidity of 2.01 g/100 mL; P6-E, cultivated for six days in GYP3, increased acidity of 0.91 g/100 mL), respectively.

### 3.2. Proteomic Analysis

#### 3.2.1. Identification and PCA of Proteins in Different Samples

The 4D label-free quantitative proteomics was used to further reveal the changes in protein abundance of *Acetobacter pasteurianus* grown under different concentrations of substrate acetic acid. A total of 17,182 unique peptides and 2094 proteins were identified in the proteome (Figure S1). Of these, 2093 had comparable quantitative information.

**Figure 2:**
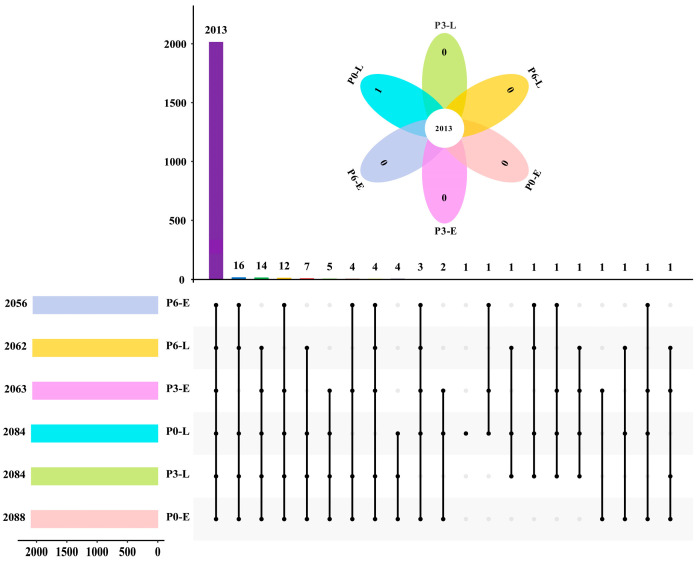
The upset diagram of the proteins in the sample. Black dots delegate the common proteins in the sample. The vertical histogram represents the number of common proteins, while the color and length of the horizontal histogram represent different samples and the number of proteins in this sample, respectively. The petal diagram in the figure shows the number of common proteins and unique proteins in all samples.

The mass spectrometry quality control results showed that most of the peptides were distributed between 7 and 20 amino acids in length, which was in line with the general pattern based on enzymatic digestion and mass spectrometry fragmentation mode (Figure S2A). In addition, the distribution of the number of peptides in proteins indicates that most proteins contain more than two specific peptide fragments, which contributes to increasing the accuracy and credibility of the quantitative and qualitative results (Figure S2B). As shown in Figure 2, the number of proteins identified in each sample exceeded 2000, with 2013 proteins commonly contained in all samples. The 4D label-free quantitative proteomics technique applied in this study greatly increased the quantity of proteins identified in *Acetobacter pasteurianus* compared to the previous research, which was 1386 proteins [17]. In another earlier study, 2289 proteins were identified in *Brucella abortus* using a label-free quantification proteomic technique [25]. Variations in protein amounts may be attributed to differences in sample types, environmental stresses, detection techniques, and so on.

**Figure 3:**
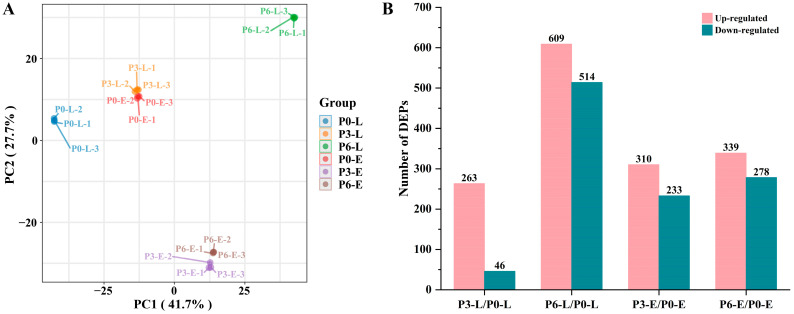
Principal component analysis (**A**) based on relative quantitative values of proteins in different samples (three biological replicates were set up for each sample) and the number of differentially expressed proteins (**B**) in each comparison group.

The PCA was performed based on the relative quantitative values of all samples, and visualized PCA plots were drawn. As shown in Figure 3A, the variance contribution of the first principal component (PC1) was 41.7% and that of the second principal component (PC2) was 27.7%, with a total explanation of 69.4%, indicating that the constructed model was plausible. As can be seen from the PCA plot, the arrangements of P0-L, P3-L, and P6-L were dispersed, whereas the arrangements of P3-E and P6-E were more clustered. The protein expression of *Acetobacter pasteurianus* in the lag phase was significantly affected by the starting acidity, and with the increase of the starting acidity, the types and relative contents of protein expression showed a stepwise change. And when the starting acidity reached a certain concentration, the metabolism of the bacterium in the exponential phase basically stabilized. Moreover, the replicates within each subgroup were substantially clustered together, indicating that the quantitative results of the biological replicates were statistically consistent. Meanwhile, relative standard deviation (RSD) analysis displayed that the mean RSD value of three biological replicates in the six groups was <0.2, which also demonstrated a high reproducibility (Figure S3).

#### 3.2.2. Screening of DEPs in Different Samples

**Figure 4:**
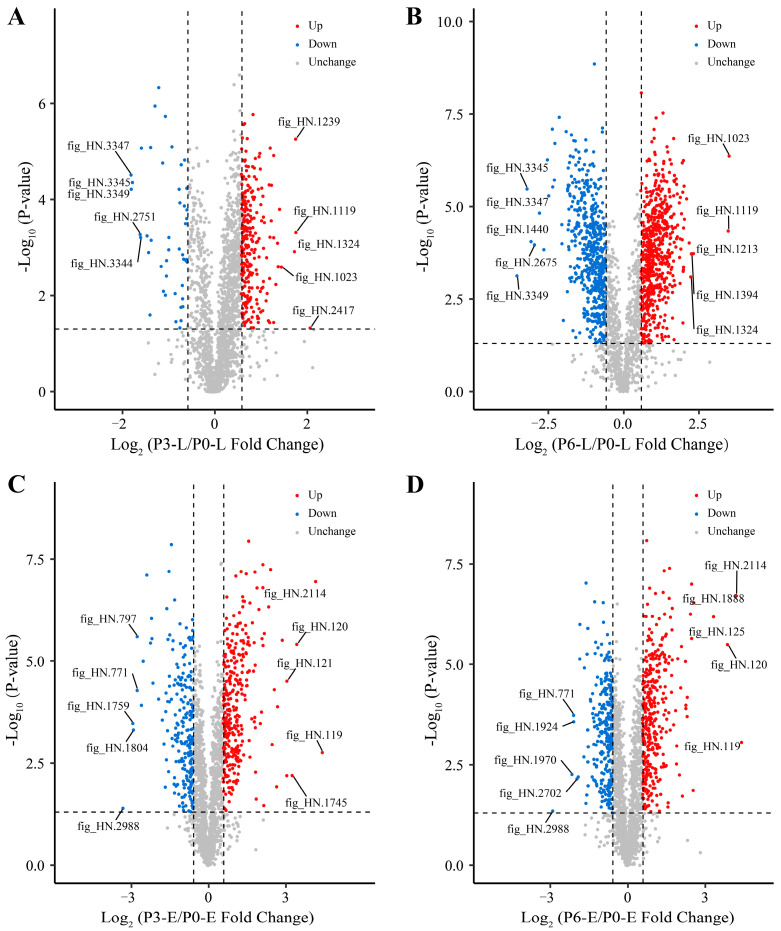
Volcano diagram of differentially expressed proteins in P3-L/P0-L (**A**), P6-L/P0-L (**B**), P3-E/P0-E (**C**), and P6-E/P0-E (**D**). In the volcano diagram, red dots indicated significant up-regulated, blue dots indicated significant down-regulated, and grey dots indicated no significant change. Detailed proteins annotation and expression data were displayed in Table S3.

The investigation of differential protein expression can screen key proteins of relevant pathways and reveal the molecular mechanism of life activities under certain conditions [26]. The changes in differential expression of more than 1.5 and less than 0.67 (*p* < 0.05) were used as thresholds of FC for significant up-regulated and down-regulated, respectively. As depicted in Figure 3B, there were 309 DEPs (up-regulated: 263; down-regulated: 46) in P3-L/P0-L and 1123 DEPs (up-regulated: 609; down-regulated: 514) in P6-L/P0-L during the lag phase of *Acetobacter pasteurianus* growth (Table S3). In the exponential phase, there were 543 DEPs (up-regulated: 310; down-regulated: 233) in P3-E/P0-E and 617 DEPs (up-regulated: 339; down-regulated: 278) in P6-E/P0-E (Table S3). The number of DEPs that were up-regulated was higher than down-regulated in all of the treatment groups compared to the control group without acetic acid. The volcano plot demonstrated the distribution and changes of all DEPs in each comparison group, where the top five DEPs of FC value were labeled (Figure 4A–D). In each of the comparison groups, the proteins with large differential changes were not quite the same, suggesting that *Acetobacter pasteurianus* had its own specific protein regulatory ways in different acid stress environments.

#### 3.2.3. Subcellular Localization of DEPs in Different Samples

**Figure 5:**
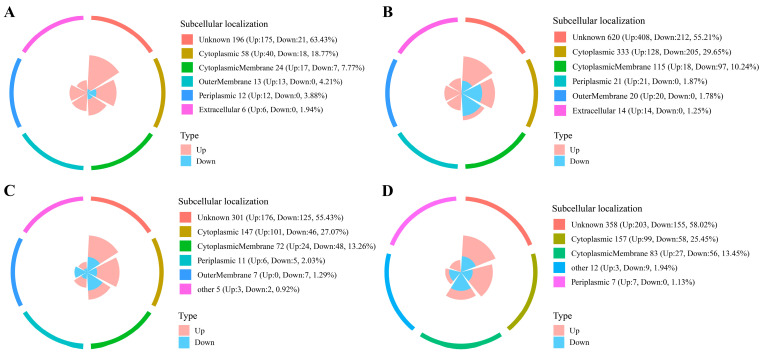
Classification rose diagram of subcellular structure annotation of differentially expressed proteins ((**A**): P3-L/P0-L, (**B**): P6-L/P0-L, (**C**): P3-E/P0-E, (**D**): P6-E/P0-E). Pink represented up-regulated differential expressed proteins and light blue represented down-regulated differential expressed proteins.

Subcellular localization refers to the localization of molecules within a cell, which means determining where the molecule is located within the cell. Proteins must be in a specific subcellular structure in order to fulfill their correct and stable biological function [27]. The subcellular localization of DEPs at the different substrate acetic acid concentrations was depicted in Figure 5A–D. As a whole, the top three major positions of subcellular localization were unknown > cytoplasmic > cytoplasmic membrane, which accounted for more than 7% of the total DEPs in each sample. Furthermore, the outer membrane, periplasmic and extracellular also occupied a certain proportion in their respective samples. The cytoplasm is the main site of life activities and contains a wide range of substances such as the ribosome, a variety of enzymes and intermediate metabolites, various nutrients, and monomers of macromolecules. The cell membrane is mainly composed of lipids and proteins, with selective exchange of substances, absorption of nutrients, discharge of metabolic waste, secretion and transport of proteins, and other physiological functions [28]. Some of the proteins in the cytoplasm and cell membrane of *Acetobacter pasteurianus* were regulated under different acidic environmental stresses to meet their survival and growth needs. Notably, the location of some proteins in the cell was unclear. In the subcellular localization of fat globular membrane proteins in human milk, nuclear, plasma membrane, and cytoplasmic also occupied dominant positions [29].

#### 3.2.4. GO Enrichment Analysis of DEPs in Different Samples

**Figure 6:**
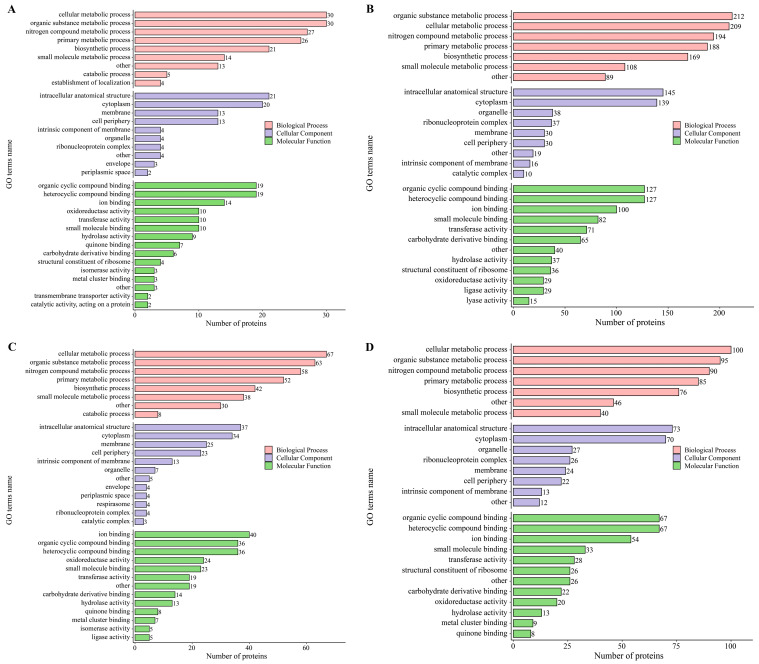
GO term enrichment analysis of differentially expressed proteins in the four comparisons ((**A**): P3-L/P0-L, (**B**): P6-L/P0-L, (**C**): P3-E/P0-E, (**D**): P6-E/P0-E).

GO is used to describe various properties of genes and proteins. GO annotations are classified into three main categories: biological processes (BP), cellular components (CC), and molecular functions (MF) [29]. GO terms that were significantly (*p* < 0.05) enriched in each comparison group were selected for analysis. In all comparison groups (Figure 6A–D), the DEPs were mainly common annotated to the ‘cellular metabolic process’, ‘organic substance metabolic process’, ‘nitrogen compound metabolic process’, ‘primary metabolic process’, ‘biosynthetic process’ in BP, and ‘intracellular anatomical structure’, and ‘cytoplasm’ in CC; ‘organic cyclic compound binding’, ‘heterocyclic compound binding’, ‘ion binding’ in MF. Moreover, ‘membrane’, ‘cell periphery’, ‘organelle’, ‘ribonucleoprotein complex’ in CC; ‘oxidoreductase activity’, ‘transferase activity’, ‘small molecule binding’, ‘carbohydrate derivative binding’ in MF were enriched for DEPs in different comparative groups, respectively. The results showed that most of the DEPs were involved in metabolic processes, cellular metabolic processes, and substance binding, indicating that acid treatment mainly affected the physiological metabolism of the strain. However, the strains in different comparison groups had their own special regulatory mechanisms to further adapt to their own acidic environment. Interestingly, the amount of DEPs enriched in the GO terms of the exponential phase was significantly higher than that of the lag phase. And the number of DEPs enriched in GO terms of 3% substrate acetic acid treatment was significantly lower than that of 6% substrate acetic acid treatment. This result indicated that the strain belongs to the stage of adapting to the new environment when it is in the lag phase, and all aspects of metabolism and regulation levels are still at a low level. In the exponential phase, the strain had fully adapted to the new environment and was able to actively carry out metabolic reactions and protein regulation to meet its growth needs in the face of high acid stress.

#### 3.2.5. KEGG Pathway Analysis of DEPs in Different Samples

**Figure 7:**
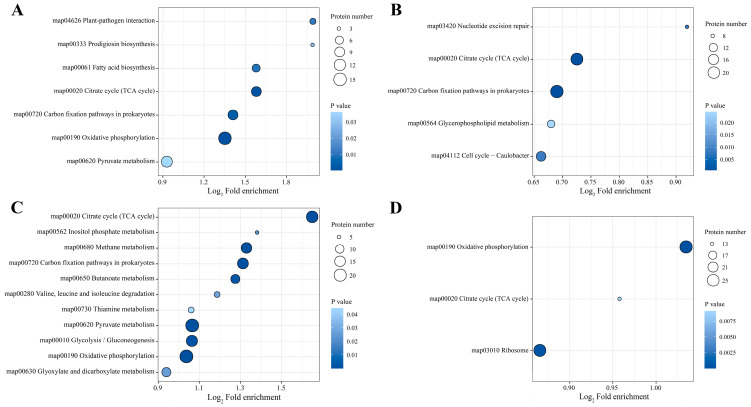
KEGG pathway enrichment analysis of differentially expressed proteins in the four comparisons ((**A**): P3-L/P0-L, (**B**): P6-L/P0-L, (**C**): P3-E/P0-E, (**D**): P6-E/P0-E). The vertical axis was the KEGG pathway description information, the horizontal axis was the degree of functional enrichment (Fold enrichment) after Log_2_ conversion, the larger the value indicated the higher degree of enrichment; the color of the dots indicated the enrichment significance of the *p* value, the darker the blue represented the stronger the significance of enrichment; the size of the dots indicated the number of differential expressed proteins in the KEGG pathway, the larger the dots indicated that there were more of the differential expressed proteins in the KEGG pathway.

KEGG is a comprehensive database that integrates genomics, biochemistry, and systemic functional genomics information and is a powerful tool for metabolic analysis and metabolic network studies in organisms. In order to thoroughly analyze the acid tolerance mechanism of *Acetobacter pasteurianus* at different substrate acetic acid concentrations, KEGG pathway enrichment analysis was performed on DEPs. The 7, 5, 11, and 3 significantly (*p* < 0.05) enriched KEGG pathways were screened in P3-L/P0-L, P6-L/P0-L, P3-E/P0-E, and P6-E/P0-E, respectively, and the tricarboxylic acid cycle (TCA cycle) was the common pathway in the four comparison groups (Figure 7). In P3-L/P0-L, the pathways that were enriched to a higher degree were oxidative phosphorylation, the TCA cycle, carbon fixation pathways in prokaryotes, pyruvate metabolism and fatty acid biosynthesis (Figure 7A). In P6-L/P0-L, there were a higher enrichment of pathways such as the carbon fixation pathways in prokaryotes, the TCA cycle, cell cycle-caulobacter and glycerophospholipid metabolism (Figure 7B). These pathways were involved in energy metabolism, carbohydrate metabolism, cell growth and death, and lipid metabolism of the organism. We speculated that proteins associated with energy metabolism, material metabolism, and cellular differentiation were regulated to adapt to the stress of the substrate acetic acid during the lag phase of strain. The TCA cycle, oxidative phosphorylation, pyruvate metabolism, glycolysis/gluconeogenesis, methane metabolism, and carbon fixation pathways in prokaryotes were the major KEGG pathways in P3-E/P0-E (Figure 7C). The ribosome, oxidative phosphorylation, and the TCA cycle are the major KEGG enrichment pathways in P6-E/P0-E (Figure 7D). These pathways were associated with energy metabolism, carbohydrate metabolism, and translation. This result suggested that a large number of proteins participated in energy and material metabolism to resist acetic acid stress and produce acetic acid during the exponential phase of the strain.

#### 3.2.6. PPI Network Analysis of DEPs in Different Samples

**Figure 8:**
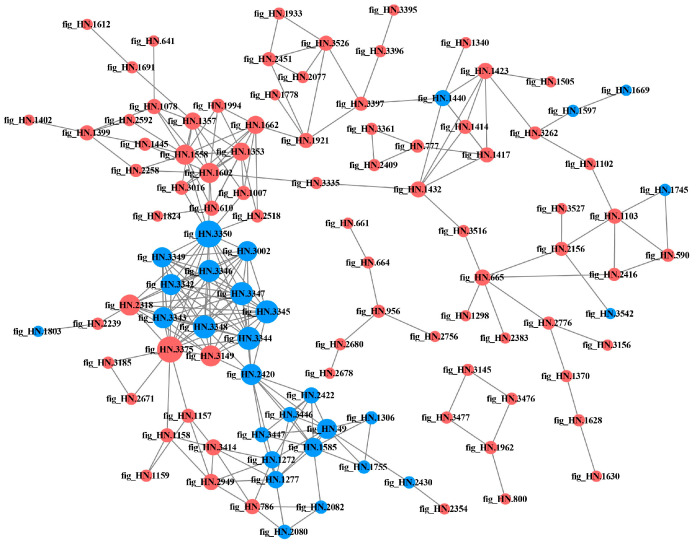
Protein–protein interaction networks of differentially expressed proteins in P3-L/P0-L. Red and blue colors indicated up-regulated and down-regulated proteins, respectively. The greater the connectivity of a differentially expressed protein, the larger the dot where that protein was located.

**Figure 9:**
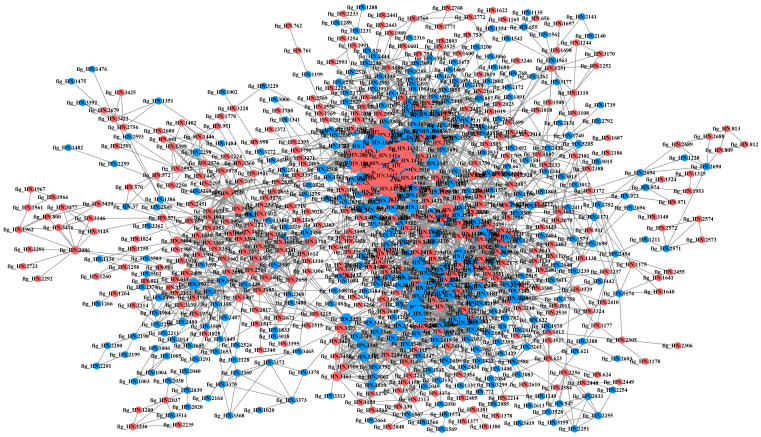
Protein–protein interaction networks of differentially expressed proteins in P6-L/P0-L. Red and blue colors indicated up-regulated and down-regulated proteins, respectively. The greater the connectivity of a differentially expressed protein, the larger the dot where that protein was located.

**Figure 10:**
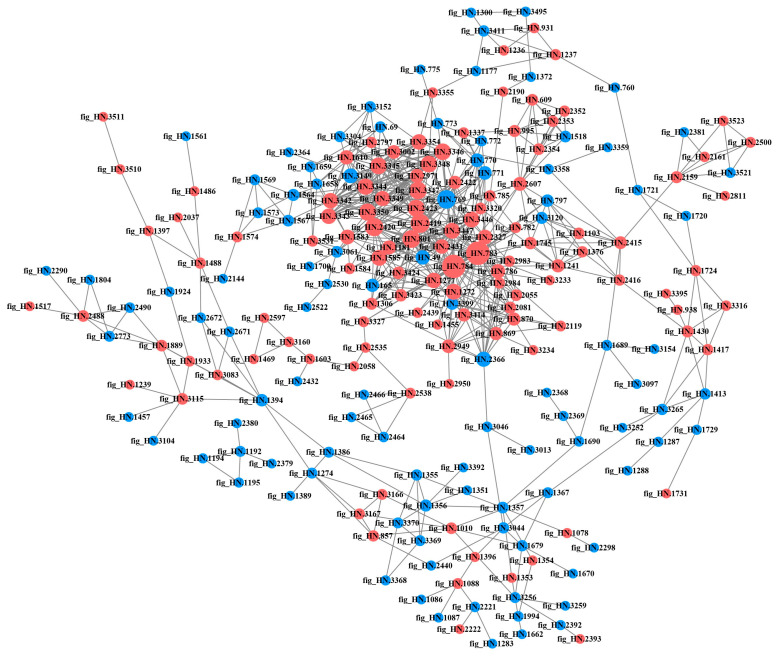
Protein–protein interaction networks of differentially expressed proteins in P3-E/P0-E. Red and blue colors indicated up-regulated and down-regulated proteins, respectively. The greater the connectivity of a differentially expressed protein, the larger the dot where that protein was located.

**Figure 11:**
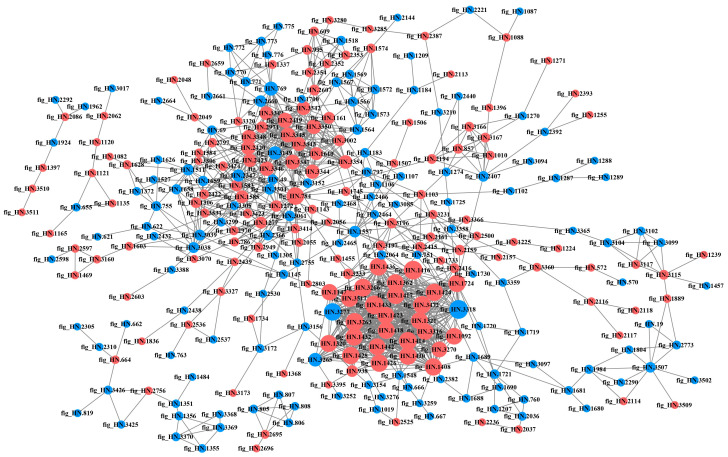
Protein–protein interaction networks of differentially expressed proteins in P6-E/P0-E. Red and blue colors indicated up-regulated and down-regulated proteins, respectively. The greater the connectivity of a differentially expressed protein, the larger the dot where that protein was located.

The PPI is involved in almost all the important biological processes within the living body and play a crucial role in the fundamental life processes of the cell [30]. Highly aggregated proteins often exhibit similar functions [31], while proteins with a high degree of connectivity may be key points for influencing metabolic reactions [27]. The DEPs in each comparison group were compared with the STRING database, and protein interaction network maps were drawn by screening the proteins with closer interactions (Figure 8, Figure 9, Figure 10 and Figure 11). The protein network in P6-L/P0-L was the most complex and involved the highest number of proteins among all comparison groups, suggesting that the strain actively regulated a large number of proteins in its metabolic activities to face the stress of the high-acid environment. In the lag phase, several of the most highly connected (degree > 10) proteins (fig_HN.3350, fig_HN.3344, fig_HN.3345, fig_HN.3347, fig_HN.3348, fig_HN.3346, fig_HN.3342, fig_HN.3343, fig_HN.2420, fig_HN.2318, fig_HN.3149) in P3-L/P0-L belonged to the oxidative phosphorylation pathway and were involved in the energy metabolism of organisms (Figure 8). Moreover, fig_HN.3375 (acyl carrier protein, ACP) belonging to fatty acid synthesis pathways had the highest connectivity among the up-regulated proteins. The study had shown that ACPs were major participants in fatty acid biosynthesis in bacterial cells and were closely linked to membrane lipid and energy metabolism [32,33]. We speculated that the strain may face 3% substrate acetate stress during the lag phase by regulating proteins related to energy metabolism and cell membrane composition. In P6-L/P0-L, most of the DEPs (fig_HN.1432, fig_HN.3051, fig_HN.1417, fig_HN.1422, fig_HN.1443, and so on) with higher connectivity (degree > 50) were belongs to ribosomal proteins and were significantly up-regulated (Figure 9). However, these DEPs were not significantly enriched in the KEGG pathway of the ribosome.

In the exponential phase, DEPs (fig_HN.783, fig_HN.784, fig_HN.2419, fig_HN.2420, fig_HN.801, fig_HN.3347, fig_HN.3350, fig_HN.2423) with higher connectivity (degree > 20) in P3-E/P0-E were affiliated with pyruvate metabolism, the TCA cycle and oxidative phosphorylation pathways and were significantly up-regulated (Figure 10). However, in P6-E/P0-E, the most highly connected (degree > 19) DEPs (fig_HN.1416, fig_HN.1433, fig_HN.3263, fig_HN.3345, fig_HN.3347, fig_HN.3350, fig_HN.784, fig_HN.2420) belonged to the ribosomal pathway, followed by oxidative phosphorylation, pyruvate metabolism, and TCA cycle pathways, all of which were significantly up-regulated in DEPs (Figure 11). The aggregation of significantly down-regulated proteins was more dispersed compared to up-regulated proteins, suggesting that up-regulated proteins may play a more important role in the acid-tolerance properties of the strains. Nevertheless, the positive effects of down-regulated proteins on acid stress should not be overlooked.

## 4. Discussion

**Figure 12:**
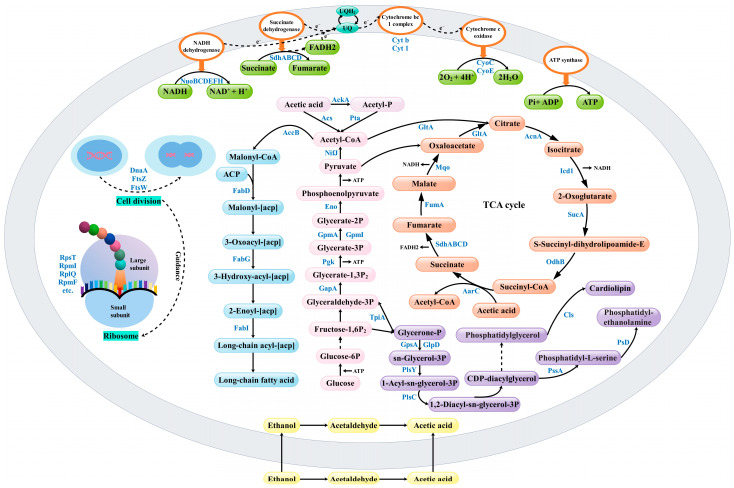
Illustration of the remarkable metabolic pathways and regulation in which significantly differentially expressed proteins were involved. Detailed protein annotation and expression data were displayed in Table 1.

The processes of lipid metabolism, the TCA cycle, glycolysis, pyruvate metabolism, energy metabolism, and cell cycle regulation as well as translation were found to be closely related to the acid resistance of *Acetobacter pasteurianus* by GO enrichment analysis and KEGG pathway analysis and the important proteins were identified from the above processes as shown in Table 1. The illustration of relevant metabolic pathways and regulation were diagrammed based on the discussion (Figure 12).

### 4.1. Fatty Acid Biosynthesis and Glycerophospholipid Metabolism

When subjected to environmental stress, the cell membranes of microorganisms are the first to be affected. The main components of cell membranes are lipids, and fatty acid biosynthesis was an important source of lipids. When the intracellular fatty acid synthesis pathway was enhanced, it was beneficial to maintain the fluidity of cell membranes and improve the stress resistance of organisms [34,35]. Glycerophospholipids were composed of two fatty acid chains, a glycerol unit, and a phosphate group, and their structure and distribution determine the properties and related functions of cell membranes [33]. *Escherichia coli* resists exogenous octanoic acid stress by altering the composition and relative abundance of membrane fatty acids [36]. *Lactobacillus casei* resisted the damage of lactic acid to the cell membrane by increasing the proportion and average chain length of unsaturated fatty acids and the fluidity of the membrane [37]. *Gluconacetobacter europaeus* V3 showed a 7.3-fold increase in phosphatidylglycerol and a 2.7-fold decrease in phosphatidylethanolamine in the presence of 3% (*v*/*v*) acetic acid [38]. In the lag phase of *Acetobacter pasteurianus*, we found that key proteins acetyl-CoA carboxylase (fig_HN.2949 and fig_HN.3414), malonyl CoA-acyl carrier protein transacylase (FabD), 3-oxoacyl-ACP reductase (FabG), and enoyl-ACP reductase (FabI) associated with type II fatty acid biosynthesis were significantly up-regulated in the presence of 3% acetic acid (Table 1). With the exception of FabI, these DEPs were also significantly up-regulated in 6% acetic acid and had higher folding changes value (Table 1). In particular, most of the DEPs associated with glycerophospholipid metabolism were down-regulated at 6% acetic acid concentration, including GlpD, Cls, Psd, PlsY, PlsC, and PssA (Figure 12). However, the expression of proteins related to lipid metabolism was not evident in the exponential phase of the strain. The results showed that enhanced fatty acid biosynthesis was an essential pathway for the strain to resist high concentrations of acetic acid stress when in the lag phase. Nevertheless, high concentrations of acetic acid stress decreased the metabolism of glycerophospholipids, reducing the damage.

### 4.2. Acetic Acid Assimilation and the TCA Cycle

Acetic acid assimilation was considered to be one of the mechanisms of acid resistance in AAB. There are three ways of assimilating acetic acid after it enters the cell: (1) Excretion of acetic acid out of the cell by AatA, a special ABC transporter protein in AAB [4]. (2) Conversion of acetic acid to acetyl-CoA under the action of acetate kinase (AckA), phosphotransferase (Pta) and acetyl-CoA synthase (Acs), and assimilation through the TCA cycle. This process not only provides energy for acetic acid fermentation, but also reduces the amount of acetic acid in the cell and attenuates the toxic effect of acetic acid on the cell [11]. (3) succinyl-CoA: acetate CoA transferase (AarC) catalyzes the conversion of succinyl-CoA and acetic acid to acetyl-CoA and succinic acid, showing a special TCA cycle [13]. In this study, Pta was up-regulated in all four comparison groups, and AckA and Acs were up-regulated in the lag phase of strain, with no significant change in the exponential phase. During the lag phase, the citrate synthase (GltA), isocitrate dehydrogenase (Icd1), 2-oxoglutarate dehydrogenase (SucA), AarC, succinate dehydrogenase (SdhAD), and fumarate hydratase (FumA), which were involved in the TCA cycle, were down-regulated in 3% acetic acid. The other down-regulated proteins were also taken part in the TCA cycle at 6% acetic acid concentration, including aconitate hydratase (AcnA), pyruvate dehydrogenase (PdhA), dihydrolipoyl dehydrogenase (LpdA) and malate dehydrogenase (Mqo), and so on (Table 1 and Figure 12). These proteins were up-regulated during the exponential phase of the strain compared to the lag phase and the fold change value of AarC was the highest among these proteins. Proteins associated with acetic acid assimilation were down-regulated due to the dual stress of the strain being in an acidic environment and a lag period, and their resistance was relatively weak. The higher the substrate acetic acid concentration, the more proteins were down-regulated. However, during the exponential phase, many proteins associated with the TCA cycle were up-regulated to assimilate acetic acid, attenuating the toxicity of acetic acid to cells. Among them, the TCA cycle, in which AarC was involved, was one of the key acid tolerance mechanisms.

### 4.3. Pyruvate Metabolism and Glycolysis

Pyruvate plays a pivotal role in linking the three major metabolisms of glucose, fatty acids, and amino acids through acetyl-CoA. For example, pyruvate can be oxidized to the intermediate metabolite acetyl-CoA by pyruvate ferredoxin/flavodoxin oxidoreductase, which entered the TCA cycle to provide intermediate metabolites and energy for bacterial growth [11]. Under the action of acetyl-CoA carboxylase, acetyl-CoA was catalyzed into malonyl-CoA and participated in the biosynthesis of fatty acids [39]. Pyruvate can be converted to alanine by transaminases into amino acid metabolism [40]. In this study, pyruvate ferredoxin/flavodoxin oxidoreductase (fig_HN.783 and fig_HN.784) was down-regulated in 6% substrate acetic acid during the lag phase of the strain and up-regulated when the strain was in the exponential phase (Table 1 and Figure 12). This change remained basically consistent with the trend of regulation of related proteins in the TCA cycle of the strain under 6% acetic acid stress. Proteins related to amino acid metabolism were not significantly or heavily enriched in our study, but their role in microbial resilience was still not negligible.

In the glycolysis pathway, glucose is converted to pyruvate by a series of enzymes and a certain amount of energy is released [41]. During the exponential phase, triosephosphate isomerase (TpiA), glyceraldehyde-3-phosphate dehydrogenase (Gap), phosphoglycerate kinase (Pgk), histidine phosphatase family protein (GpmB), 2,3-bisphosphoglycerate-independent phosphoglycerate mutase (GpmI), and enolase (Eno), which were involved in the glycolysis pathway, were significantly up-regulated under 3% acetic acid (Table 1 and Figure 12). This result suggests that the strain may resist acid stress and produce acetic acid by enhancing glucose and pyruvate metabolism, which further promote energy release and material conversion.

### 4.4. Energy Metabolism

Energy metabolism is a central issue in the metabolism of living organisms and the maintenance of life. Oxidative phosphorylation was a biochemical process, a coupling reaction in which the energy released during the oxidation of substances in the body was supplied through the electron transport chain to adenosine diphosphate (ADP) and inorganic phosphate for the synthesis of adenosine-triphosphate (ATP) and was a meaningful source of energy in living organisms [42]. Nicotinamide adenine nucleotide (NADH) and flavin adenine dinucleotide (FADH2) produced in the TCA cycle were oxidized for electron transfer in the electron transport chain [43]. Five enzymes, including NADH dehydrogenase, succinate dehydrogenase, ubiquinol-cytochrome c reductase, cytochrome c oxidase, and ATP synthase, dominated the oxidative phosphorylation process [43]. It had been shown that genes related to oxidative phosphorylation were also significantly up-regulated in the mid and final stages of industrial production of high concentrations of AAB [44]. In this research, NADH dehydrogenase (NuoBCDEGHI) and succinate dehydrogenase (SdhA, SdhD) were down-regulated in 3% substrate acetic acid when the strain was in the lag phase; NuoB, NuoDEGHI, NuoM, SdhABCD, cytochrome c oxidase (CyoE and CyoC), and cytochrome c reductase (Cyt b and Cyt 1) were down-regulated in the presence of 6% substrate acetic acid (Table 1 and Figure 12). During the exponential phase, NuoBCDEGHI and SdhABCD were up-regulated in both comparison groups, while Cyt b and Cyt 1 were down-regulated (Table 1). In addition, CyoE and CyoC were down-regulated with 6% substrate acetic acid. The results showed that the energy metabolism pathway was weakened during the lag phase of the strain, and the higher the acidity, the more proteins were down-regulated, which might be due to the inhibitory effect of high concentrations of acetic acid on the enzyme activity. During the exponential phase of the strain, the microbial metabolic response was accelerated, and the energy metabolic pathway was strengthened to meet the dual tasks of acid production and acid tolerance. Despite being in the exponential phase, some proteins may not be able to tolerate the 6% starting acetic acid and their activities are inhibited or even inactivated.

### 4.5. Cell Cycle Regulation and Translation

The cell cycle is the entire process that a cell undergoes from the beginning of the completion of one division to the end of the next. This process included DNA replication, chromosome segregation, establishment of the division plane, cytokinesis, and all the regulatory pathways that coordinate the processes [45]. The FtsZ and FtsW have been reported to be key proteins of bacterial cell division. [46,47]. In our study, in the presence of 6% substrate acetic acid, the cell cycle-caulobacter KEGG pathway was significantly enriched in the lag phase. Of these, the proteins FtsZ, FtsQ, and FtsW related to cell division were significantly up-regulated, and the protein DnaA related to DNA replication was significantly down-regulated (Table 1 and Figure 12). This phenomenon echoes the transcriptome results of *Acetobacter pasteurianus* in response to high acid stress [9]. The results suggest that the intensified cell division process may be a potential mechanism of acid tolerance in *Acetobacter pasteurianus*, but its detailed regulatory mechanism needs to be further investigated.

The ribosome is the molecular machinery for protein synthesis in the cell and plays a crucial role in the regulation of protein synthesis [48]. It was reported that high expression of ribosomal proteins helped Cantaloupe to cope with chilling stress during the early stage of cold storage [26]. In our study, the 26 DEPs were significantly enriched in the ribosomal KEGG pathway in the exponential phase of the strain under 6% acetic acid stress, of which 25 (RpsT, RpmI, RplQ, etc.) were up-regulated and 1 (RpmF) was down-regulated (Table S2 and Figure 12). Previous transcriptomic data showed that many ribosomal protein-coding genes were significantly up-regulated under high acid stress [9]. These transcriptome results were consistent with our studies in proteomics. We speculated that the strain may resist high acid stress by increasing protein synthesis and translation during the exponential phase.

## 5. Conclusions

The 4D label-free relative quantitative proteomics analysis was utilized to display the protein profiles of *Acetobacter pasteurianus* under different concentrations of substrate acetic acid. There were more significantly up-regulated differential proteins than down-regulated in the presence of 3% and 6% substrate acetic acid. The differentially expressed proteins were major involved in gene ontology terms of metabolic processes, cellular metabolic processes, and substance binding. When acetic acid fermentation was initiated with a certain concentrations of the substrate acetic acid, proteins related to the pathways of fatty acid biosynthesis, glycerophospholipid metabolism, pyruvate metabolism, the TCA cycle, and energy metabolism were significantly differentially expressed of *Acetobacter pasteurianus* to produce and tolerate acid. In addition, the enhancement of cell cycle regulation and protein translation may be a potentially effective pathway to resist high acid stress. This study contributes to the exploration of new potential acid tolerance mechanisms of *Acetobacter pasteurianus* under different concentrations of substrate acetic acid from 4D label-free relative quantitative proteomics analysis.

## Figures and Tables

**Table 1:** Overview of important proteins in each comparative group.

Protein Accession	Protein Description	Gene Name	P3-L/P0-L Fold Change	Type	P6-L/P0-L Fold Change	Type	P3-E/P0-E Fold Change	Type	P6-E/P0-E Fold Change	Type
fig_HN.783	Pyruvate ferredoxin/flavodoxin oxidoreductase	APA01_14730	0.73		0.39	down	1.89	up	1.29	
fig_HN.784	Pyruvate ferredoxin/flavodoxin oxidoreductase	nifJ	0.69		0.37	down	2.32	up	1.61	up
fig_HN.786	Phosphate acetyltransferase	pta	1.52	up	1.78	up	2.06	up	1.65	up
fig_HN.787	Acetate kinase	ackA	0.83		0.42	down	1.25		0.95	
fig_HN.1094	Chromosomal replication initiator protein	dnaA	0.74		0.37	down	0.86		1.43	
fig_HN.1095	Chromosomal replication initiator protein DnaA	dnaA	0.74		0.40	down	0.99		1.28	
fig_HN.1103	Triosephosphate isomerase	tpiA	1.61	up	2.30	up	2.31	up	1.95	up
fig_HN.1114	Dihydrolipoyl dehydrogenase	lpdA	1.09		0.63	down	0.72		0.67	
fig_HN.1135	Phospholipase	cls	0.93		0.45	down	1.18		1.53	up
fig_HN.1158	Malonyl CoA-acyl carrier protein transacylase	fabD	1.70	up	2.58	up	1.23		1.39	
fig_HN.1159	3-oxoacyl-[acyl-carrier-protein] reductase	fabG	1.61	up	2.31	up	1.60	up	1.38	
fig_HN.1181	Probable malate:quinone oxidoreductase	mqo	0.83		0.56	down	1.59	up	1.30	
fig_HN.1209	2-nitropropane dioxygenase	fabI	1.50	up	1.44		0.81		0.66	down
fig_HN.1241	Enolase	eno	1.26		1.25		2.18	up	1.35	
fig_HN.1272	Succinyl-CoA:acetate CoA transferase	aarC	0.53	down	0.53	down	4.37	up	3.34	up
fig_HN.1277	Citrate synthase	gltA	0.38	down	0.18	down	2.95	up	2.77	up
fig_HN.1306	Isocitrate dehydrogenase (NAD+)	icd1	0.60	down	0.43	down	3.53	up	3.11	up
fig_HN.1367	2,3-bisphosphoglycerate-independent phosphoglycerate mutase	gpmI	1.23		1.33		1.65	up	1.18	
fig_HN.1544	Histidine phosphatase family protein	gpmB	0.91		0.76		2.45	up	2.15	up
fig_HN.1562	Phosphatidylserine decarboxylase proenzyme	psd	1.09		0.66	down	0.62	down	0.81	
fig_HN.1563	CDP-diacylglycerol--serine O-phosphatidyltransferase	pssA	1.22		0.59	down	0.80		0.76	
fig_HN.1577	1-acyl-sn-glycerol-3-phosphate acyltransferase	plsC	1.01		0.59	down	1.29		1.62	up
fig_HN.1585	2-oxoglutarate dehydrogenase	sucA	0.43	down	0.20	down	2.53	up	2.71	up
fig_HN.1595	Glycerol-3-phosphate acyltransferase	plsY	0.85		0.44	down	0.78		0.84	
fig_HN.1610	Proton-translocating NADH-quinone oxidoreductase	nuoM	0.62	down	0.33	down	2.16	up	1.78	up
fig_HN.1654	Acetate-CoA ligase	acs	0.89		0.50	down	0.81		0.92	
fig_HN.1755	Isocitrate dehydrogenase [NADP]	icd	0.41	down	0.18	down	0.71		1.16	
fig_HN.2080	Acetate-CoA ligase	acs	0.64	down	0.30	down	1.03		1.15	
fig_HN.2347	Cytochrome bo (3) ubiquinol oxidase	cyoC	1.10		0.64	down	0.94		0.63	down
fig_HN.2415	Glyceraldehyde-3-phosphate dehydrogenase	gap	1.36		2.16	up	1.99	up	1.72	up
fig_HN.2416	Phosphoglycerate kinase	pgk	1.59	up	1.95	up	2.54	up	1.63	up
fig_HN.2419	Succinate dehydrogenase iron-sulfur subunit	sdhB	0.77		0.53	down	2.89	up	2.42	up
fig_HN.2420	Succinate dehydrogenase flavoprotein subunit	sdhA	0.48	down	0.21	down	2.43	up	2.13	up
fig_HN.2422	Succinate dehydrogenase	sdhD	0.59	down	0.29	down	2.49	up	2.09	up
fig_HN.2423	Succinate dehydrogenase cytochrome b556 subunit	sdhC	0.57	down	0.29	down	2.68	up	2.56	up
fig_HN.2949	Biotin carboxyl carrier protein of acetyl-CoA carboxylase	FCN51_10970	2.35	up	2.38	up	2.01	up	2.08	up
fig_HN.3098	Cell division protein FtsZ	ftsZ	1.53	up	2.25	up	1.32		1.27	
fig_HN.3100	Cell division protein FtsQ	ftsQ	1.48		1.91	up	1.2		1.37	
fig_HN.3152	Cytochrome b	cyt b/cyt 1	1.07		0.60	down	0.44	down	0.41	down
fig_HN.3299	Protoheme IX farnesyltransferase	cyoE	1.03		0.61	down	0.68		0.58	down
fig_HN.3342	NADH-quinone oxidoreductase subunit B	nuoB	0.39	down	0.24	down	2.72	up	2.05	up
fig_HN.3343	NADH-quinone oxidoreductase subunit B	nuoB	0.51	down	0.34	down	2.37	up	1.77	up
fig_HN.3344	NADH-quinone oxidoreductase subunit C	nuoC	0.33	down	0.20	down	2.54	up	2.40	up
fig_HN.3345	NADH-quinone oxidoreductase subunit D	nuoD	0.29	down	0.11	down	2.07	up	2.15	up
fig_HN.3346	NAD(P)H-dependent oxidoreductase subunit E	nuoE	0.50	down	0.51	down	2.38	up	2.07	up
fig_HN.3347	NADH-quinone oxidoreductase subunit F	nuoF	0.29	down	0.15	down	2.09	up	1.99	up
fig_HN.3348	NADH-quinone oxidoreductase subunit G	nuoG	0.33	down	0.16	down	1.96	up	1.70	up
fig_HN.3349	NADH-quinone oxidoreductase subunit H	nuoH	0.29	down	0.09	down	2.43	up	1.85	up
fig_HN.3350	NADH-quinone oxidoreductase subunit I	nuoI	0.36	down	0.34	down	3.56	up	3.16	up
fig_HN.3354	NADH-quinone oxidoreductase subunit N	nuoN	0.93		0.31	down	2.22	up	2.12	up
fig_HN.3414	Biotin carboxyl carrier protein of acetyl-CoA carboxylase	accB	1.64	up	3.42	up	1.73	up	2.05	up
fig_HN.3423	Aconitate hydratase	acnA	0.67		0.42	down	2.53	up	2.19	up
fig_HN.3424	Aconitate hydratase	acnA	0.70		0.47	down	2.80	up	2.37	up
fig_HN.3446	Fumarate hydratase class I	fumA	0.61	down	0.31	down	1.57	up	1.35	
fig_HN.3447	Fumarate hydratase class I	fumA	0.64	down	0.33	down	1.55	up	1.27	

Note: In each comparison group, only the proteins labelled with the type of regulation were significantly differentially expressed proteins.

## Data Availability

Data are contained within the article and Supplementary Materials.

## Supplementary Materials

The following supporting information can be downloaded at: https://www.mdpi.com/article/10.3390/foods12244471/s1, Figure S1: Overview of protein identification; Figure S2: The distribution of peptides length (A) and peptides in proteins number (B); Figure S3: The relative standard deviation (RSD) analysis in different samples. Table S1: Growth of *Acetobacter pasteurianus* in 24 h under different concentrations of the substrate acetic acid; Table S2: The proteins enriched in the ribosome KEGG pathway of each comparison group; Table S3: Differentially expressed proteins statistics.
