# Supplementary material for: Investigation of Acid Tolerance Mechanism of Acetobacter pasteurianus under Different Concentrations of Substrate Acetic Acid Based on 4D Label-Free Proteomic Analysis
Source: Foods. 2023 Dec 13;12(24):4471. doi: 10.3390/foods12244471 (PMC10742644; doi:10.3390/foods12244471)
Supplement: Supplementary file 1 [file foods-12-04471-s001.zip › Supplementary material.pdf]

Supplementary materials for:

Investigation of acid tolerance mechanism of *Acetobacter pasteurianus* under different concentrations of substrate acetic acid based on 4D label-free proteomic analysis

Tian Li[1], Xinwei Wang[1], Chunyan Li[1], Qingquan Fu[1], Xuewei Shi [1], Bin Wang[1,*]

[1] Food college, Shihezi University, Shihezi 832000, Xinjiang Uygur Autonomous Region, P. R. China.

* Corresponding authors

E-mail addresses: binwang0228@shzu.edu.cn

Tel.: 86-0993-2058093

Catalog

Supplementary Tables **1**

Table S1. Growth of *Acetobacter pasteurianus* in 24 hours under different concentrations of the substrate acetic acid..1

Table S2. The proteins enriched in the ribosome KEGG pathway of each comparison group. 2

Supplementary Figures **3**

Figure S1. Overview of protein identification. 3

Figure S2. The distribution of peptides length (A) and peptides in proteins number (B). 4

Figure S3. The relative standard deviation (RSD) analysis in different samples. 5

Supplementary Tables

Table S1. Growth of *Acetobacter pasteurianus* in 24 hours under different concentrations of the substrate acetic acid

Time (h)	OD_{600}			Increased acetic acid (g/100mL)		
	0	3%	6%	0	3%	6%
6	0.003±0.0015^{d}	0.003±0.0015^{c}	0.003±0.0010^{a}	0.02±0.0058^{d}	0.01±0.0144^{c}	0.02±0.0115^{a}
12	0.051±0.0062^{c}	0.003±0.0015^{c}	0.003±0.0012^{a}	0.13±0.0265^{c}	0.02±0.0058^{c}	0.02±0.0058^{a}
18	0.487±0.0305^{b}	0.147±0.0126^{b}	0.004±0.0010^{a}	0.45±0.0351^{b}	0.21±0.0200^{b}	0.01±0.0058^{a}
24	0.618±0.0195^{a}	0.205±0.0282^{a}	0.003±0.0020^{a}	0.50±0.0115^{a}	0.40±0.0404^{a}	0.01±0.0058^{a}

Table S2. The proteins enriched in the ribosome KEGG pathway of each comparison group.

Protein accession	Protein description	Gene name	P3-L/P0-L Fold change	Regulated type	P6-L/P0-L Fold change	Regulated type	P3-E/P0-E Fold change	Regulated type	P6-E/P0-E Fold change	Regulated type
fig_HN.1092	Small ribosomal subunit protein bS20	rpsT	1.27		1.55	up	1.49		2.54	up
fig_HN.1320	Large ribosomal subunit protein bL20	rplT	0.83		0.40	down	1.05		1.51	up
fig_HN.1321	Large ribosomal subunit protein bL35	rpmI	1.18		2.49	up	1.04		3.27	up
fig_HN.1362	Small ribosomal subunit protein bS18	rpsR	1.23		1.54	up	1.44		1.57	up
fig_HN.1408	Large ribosomal subunit protein bL17	rplQ	1.40		1.87	up	1.20		1.74	up
fig_HN.1414	Large ribosomal subunit protein uL15	rplO	1.52	up	2.10	up	1.37		1.76	up
fig_HN.1416	Small ribosomal subunit protein uS5	rpsE	1.08		0.97		1.43		2.22	up
fig_HN.1417	Large ribosomal subunit protein uL18	rplR	1.51	up	3.22	up	1.68	up	2.21	up
fig_HN.1418	Large ribosomal subunit protein uL6	rplF	1.41		2.24	up	1.29		1.58	up
fig_HN.1423	Large ribosomal subunit protein uL14	rplN	1.56	up	2.15	up	1.36		1.70	up
fig_HN.1424	Small ribosomal subunit protein uS17	rpsQ	1.27		1.71	up	1.37		1.53	up
fig_HN.1426	Large ribosomal subunit protein uL16	rplP	1.11		1.15		1.48		2.14	up
fig_HN.1428	Large ribosomal subunit protein uL22	rplV	1.12		1.46		1.26		1.70	up
fig_HN.1430	Large ribosomal subunit protein uL2	rplB	0.77		0.33	down	1.68	up	3.34	up
fig_HN.1432	Large ribosomal subunit protein uL4	rplD	1.55	up	2.03	up	1.41		1.76	up
fig_HN.1433	Large ribosomal subunit protein uL3	rplC	1.04		1.27		1.14		1.51	up
fig_HN.1436	Small ribosomal subunit protein uS12	rpsL	1.00		0.68		1.17		2.57	up
fig_HN.1442	Large ribosomal subunit protein uL10	rplJ	1.34		1.80	up	1.35		1.62	up
fig_HN.2246	Large ribosomal subunit protein bL36	rpmJ	1.43		2.73	up	2.02	up	2.61	up
fig_HN.3263	Small ribosomal subunit protein bS16	rpsP	1.30		1.74	up	1.45		1.67	up
fig_HN.3266	Large ribosomal subunit protein bL19	rplS	1.35		1.92	up	1.15		1.54	up
fig_HN.3270	Small ribosomal subunit protein bS21	rpsU	0.86		0.56	down	1.49		2.36	up
fig_HN.3277	Large ribosomal subunit protein bL32	rpmF	1.17		2.43	up	0.61	down	0.44	down
fig_HN.3316	Large ribosomal subunit protein bL28	rpmB	1.23		1.93	up	1.55	up	1.76	up
fig_HN.3472	Small ribosomal subunit protein uS9	rpsI	1.31		1.58	up	1.42		1.86	up
fig_HN.3517	Small ribosomal subunit protein uS15	rpsO	1.44		1.68	up	1.22		1.67	up

Note: In each comparison group, only the proteins labeled with the type of regulation were significantly differentially expressed proteins.

Supplementary Figures

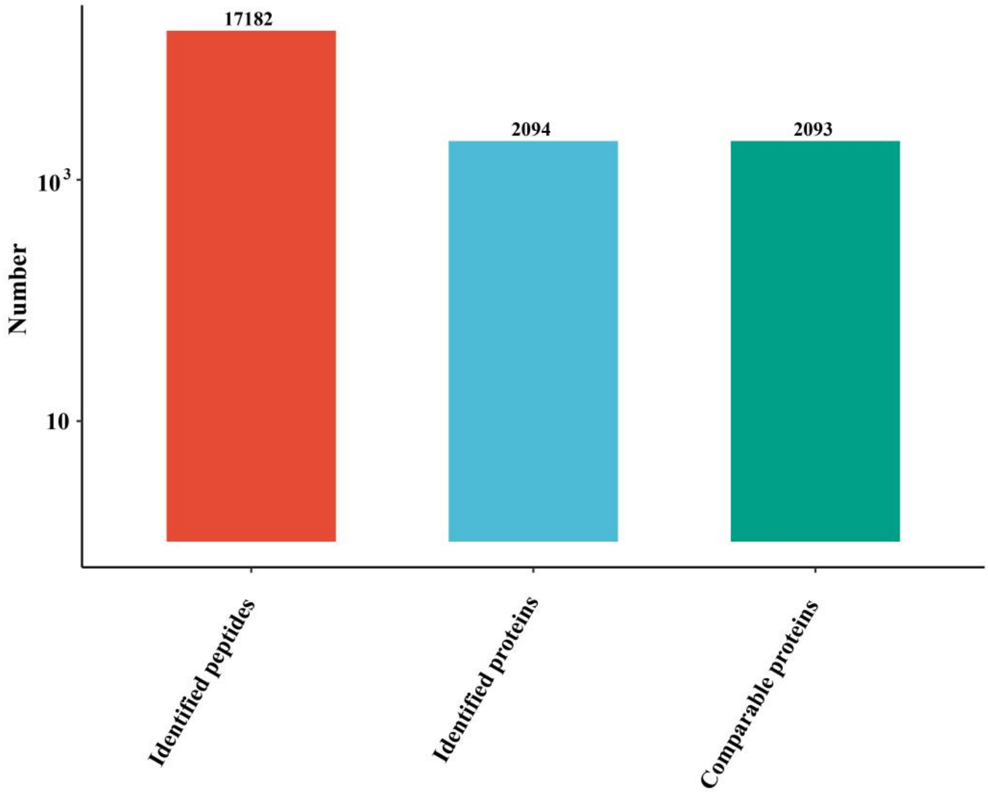

Figure S1. Overview of protein identification.

A

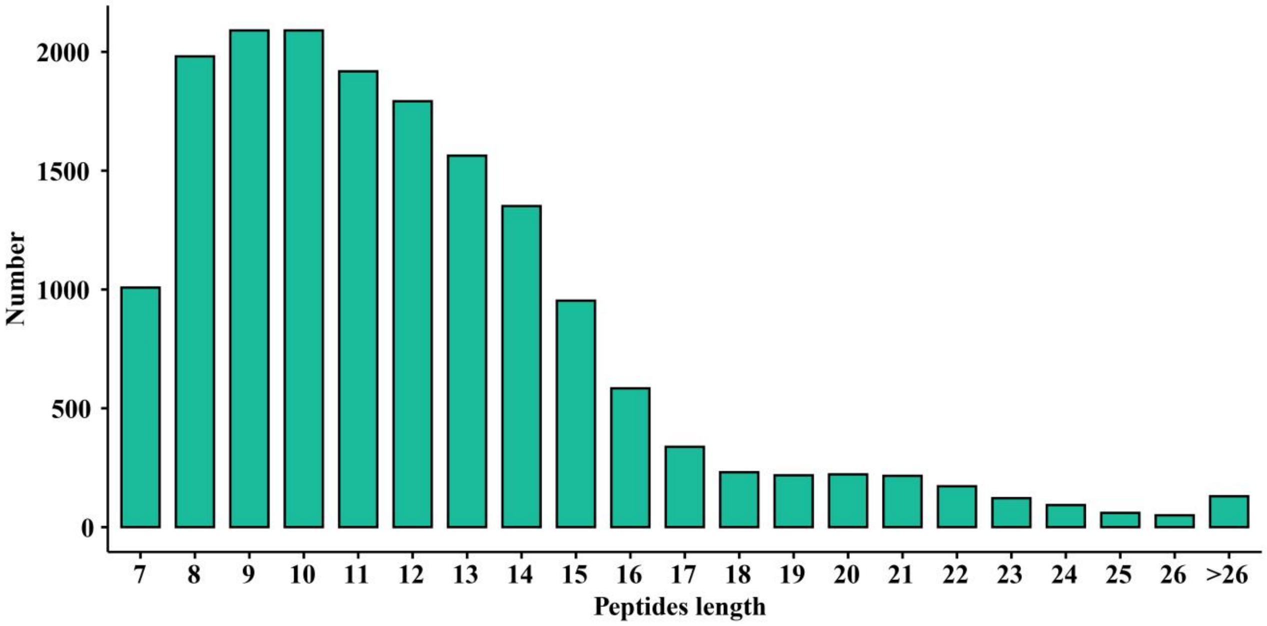

B

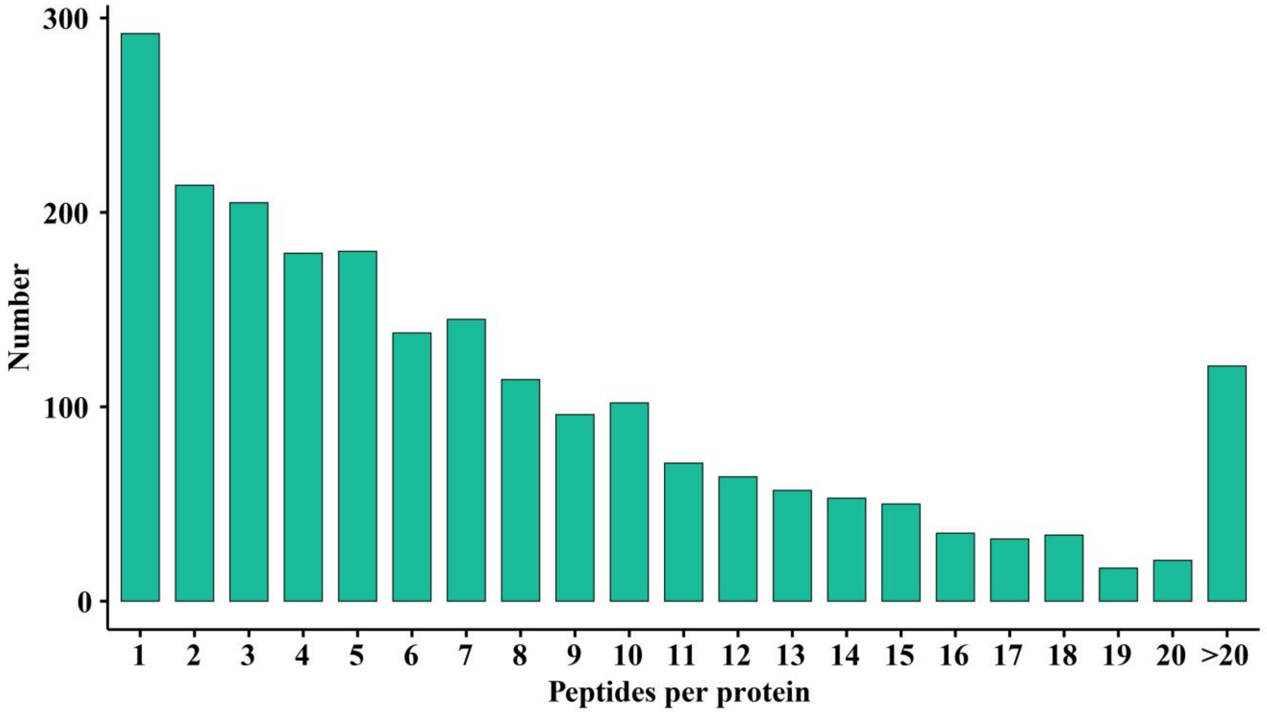

Figure S2. The distribution of peptides length (A) and peptides in proteins number (B).

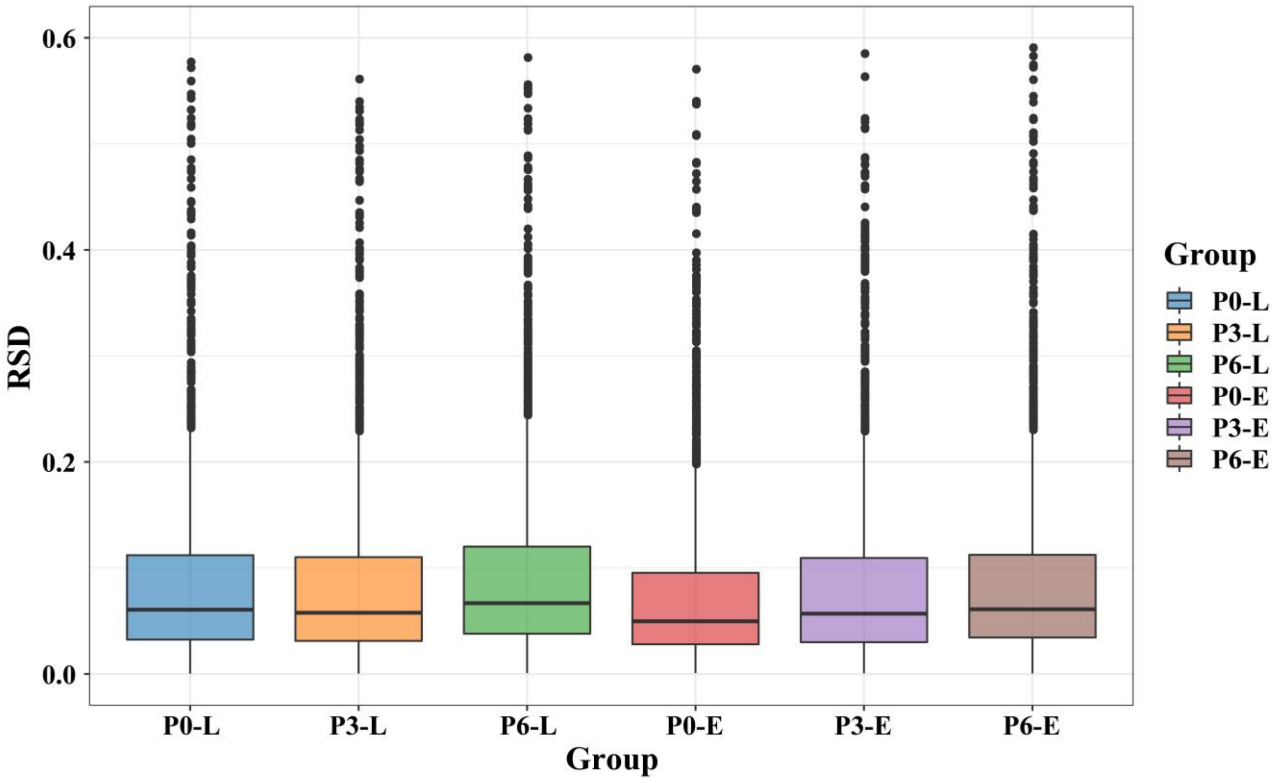

Figure S3. The relative standard deviation (RSD) analysis in different samples.
